# Comparative Transcriptional Profiling and Physiological Responses of Two Contrasting Oat Genotypes under Salt Stress

**DOI:** 10.1038/s41598-018-34505-5

**Published:** 2018-11-02

**Authors:** Bin Wu, Yarvaan Munkhtuya, Jianjiang Li, Yani Hu, Qian Zhang, Zongwen Zhang

**Affiliations:** 1 Institute of Crop Science, Chinese Academy of Agricultural Sciences (CAAS), No. 12, Zhongguancun South Street, Beijing, 100081 China; 2 Institute of Grain Crops, Xinjiang Academy of Agricultural Sciences, No. 403, Nanchang Road, Urumqi, 830091 China

## Abstract

Salinity is one of the major abiotic factors that affect productivity in oat. Here, we report a comparison of the transcriptomes of two hexaploid oat cultivars, ‘Hanyou-5’ and ‘Huazao-2’, which differ with respect to salt tolerance, in seedlings exposed to salt stress. Analysis of the assembled unigenes from the osmotically stressed and control libraries of ‘Hanyou-5’ and ‘Huazao-2’ showed that the expression of 21.92% (36,462/166,326) of the assembled unigenes was differentially regulated in the two cultivars after different durations of salt stress. Bioinformatics analysis showed that the main functional categories enriched in these DEGs were “metabolic process”, “response to stresses”, “plant hormone signal transduction”, “MAPK signalling”, “oxidative phosphorylation”, and the plant-pathogen interaction pathway. Some regulatory genes, such as those encoding MYB, WRKY, bHLH, and zinc finger proteins, were also found to be differentially expressed under salt stress. Physiological measurements also detected significant differences in the activities of POD (76.24 ± 1.07 Vs 81.53 ± 1.47 U/g FW) in the two genotypes in response to osmotic stress. Furthermore, differential expression of 18 of these genes was successfully validated using RNA-seq and qRT-PCR analyses. A number of stress-responsive genes were identified in both cultivars, and candidate genes with potential roles in the adaptation to salinity were proposed.

## Introduction

Oat is a cereal crop of global importance that is used for food, feed, and forage. Developments in the field of nutrition have led to the recognition of oats as a healthful food since the mid-1980s. Oats are known to contain soluble fibre that can help prevent heart disease, making them a popular part of the human diet^1^. Based on whether the caryopsis is tightly covered by the husk, oats can be divided into common oats and naked oats. Unlike common oats, naked oats (*Avena sativa* subsp. *nudisativa*) do not require dehulling and can therefore be used for food production as whole grains, maintaining healthful properties due to the presence of bioactive compounds in the outer layers of the caryopsis^2^. Moreover, previous studies indicate that naked oat grains have higher nutritional value than the caryopsis of husked oats and other species such as wheat or barley, including large amounts of soluble fibre, a higher oil content, and higher levels of some essential amino acids^3^. Naked oat is a dominant crop in some marginal areas of northern and western China that are affected by salinity and aridity, where it plays an important role in the local economy and environmental ecology^4^. Breeding for improved tolerance to abiotic stresses such as salt and drought is critically important for oat cultivation in China. Naked oat is a traditional crop in China, and its long-term domestication has generated an abundance of ecologically adapted cultivars. Studies on naked oat have shown considerable biological diversity among different cultivars with respect to adaptation to salt stress^5^, but the current understanding of these stress-adaptive mechanisms is relatively limited.

Next-generation sequencing (NGS) provides an efficient, cost-effective means to generate genomic resources on a large scale. NGS has proven to be an effective method for gene discovery, the identification of new molecular markers, and the generation of sequence data for the analysis of transcriptional profiles. Elucidating the molecular basis of abiotic stress tolerance in crops is facilitated by NGS-based approaches^6^. Many studies employing transcriptome analysis in both model and non-model plant species have been reported since the emergence of NGS technology. In oat, a pioneering study has begun to reveal large portions of the transcriptome in immature seeds, greatly increasing the number of expressed sequence tag (EST) sequences for the analysis of genes relevant to nutrition^7^. However, the molecular basis of abiotic stress tolerance in oat remains largely unknown.

Soil salinity stresses plants in two ways: (1) high concentrations of salts in the soil make it difficult for roots to absorb water, and (2) high concentrations of salts within plant cells can be toxic. Accordingly, plant growth responds to salinity in two phases: (1) an initial rapid phase in which the growth of young leaves is inhibited (the osmotic phase) and (2) a slower phase in which the senescence of mature leaves is accelerated (the ionic phase)^8^. Because the retardation in leaf development is largely due to the osmotic effect of the salt, elucidating the basic physiology and molecular genetics of the response to osmotic stress and the effects of the salinity responses will increase our understanding of salt stress and enable further applications in oat. Moreover, osmotic stress is also a component of the initial stages of drought stress, which similarly involve increasing the cellular concentrations of osmolytes and regulating stomatal conductance^9^.

To discover the adaptive mechanisms involved in osmotic stress tolerance in different oat cultivars, we performed RNA-seq on two oat genotypes with and without exposure to salt stress and identified a large number of salt-responsive genes that are differentially expressed between salt-tolerant and salt-sensitive cultivars. Through bioinformatic exploration and the further qRT-PCR validation of some representative mRNAs, we present a comparative analysis and functional annotation of a subset of the transcriptional profiles of two oat cultivars that differ with respect to salt tolerance. The different transcriptome expression profiles of the two oat cultivars used in this study may provide useful insights for further analysing the mechanisms that underlie salt tolerance in oat. Moreover, the genes found to be differentially expressed in the two cultivars in this study may facilitate the identification of key genes or strong salt-tolerant alleles that could potentially be suitable targets for biotechnological manipulation with the ultimate goal of improving salt tolerance in oat^10^.

## Methods

### Plant materials and growth conditions

Based on previously published preliminary screening experiments, we selected one salt-tolerant oat cultivar (‘Hanyou-5’) and one salt-sensitive cultivar (‘Huazao-2’) for transcriptome analysis under osmotic stress^5^. Sterilized seeds were allowed to germinate for 6 d on moist filter paper, then transferred to Hoagland’s nutrient solution and grown under 16 h/8 h light/dark, 25 °C/20 °C conditions. Salt treatment was carried out as described by Genc *et al*.^11^. Approximately 7 d after transplanting, when the third leaf was beginning to emerge, experimental oat seedlings were treated with 100 mm NaCl dissolved in Hoagland’s nutrient solution, and seedlings not subjected to NaCl treatment were used as the control. After exposure to 100 mm NaCl solutions for 2, 4, 8, 12, and 24 h, seedlings were harvested directly into liquid nitrogen and stored at −80 °C until their use for RNA extraction.

### Physiological index measurements

The physiological assays were carried out according to the methods described by Sunkar with minor modifications^12,13^. The relative water content (RWC) was calculated as follows: RWC (%) = (FW−DW)/(TW − DW) × 100. Oat fresh weight (FW) was obtained immediately after harvest, and the plants were then soaked in deionized water for 8 h at 4 °C. Then, the plants were quickly weighed (TW), and their dry mass (DW) was measured after oven-drying at 105 °C for 10 min followed by 80 °C for 24 h. Free proline content was measured according to a colorimetric method. The soluble sugar concentration was determined according to the anthrone method. For inorganic cation detection, after removal of the nutrient solution on the surface by washing with distilled water, the harvested samples were dried and baked to ash. Cations were extracted in Milli-Q water with vigorous shaking for 24 h. After a brief centrifugation, the supernatants were filtered using cellulose acetate filters, and the concentrations of Na^+^, K^+^, and Ca^2+^ were determined using an ion chromatograph system (SHIM-PACK IC-C3; Shimadzu, Kyoto, Japan). The superoxide dismutase activity was measured using a modified NBT method. The peroxidase activity was determined based on the change in absorbance at 470 nm due to the oxidation of guaiacol. The catalase activity was assayed by monitoring the decomposition of H_2_O_2_ by measuring the decrease in absorbance at 240 nm of a reaction mixture consisting of 50 mM potassium phosphate buffer (pH 7.0), 10 mM H_2_O_2_ and enzyme extract. The extinction coefficient of H_2_O_2_ (40 mM^−1^ cm^−1^ at 240 nm) was used to calculate the enzyme activity, which was expressed as millimoles of H_2_O_2_ per minute per gram FW. For the physiological experiments, three or more independent biological replicates of each control and each salt-treated sample were performed. Statistical analysis of the data was performed by analysis of variance using SPSS 16.0 software. Data are presented as the means and standard deviations (SD) of three replicates.

### RNA extraction and double-stranded cDNA synthesis

Total RNA was extracted from whole plants using the RNeasy Plant Mini Kit (Qiagen) according to the manufacturer’s instructions. mRNA was isolated from the qualified total RNA using the Oligotex mRNA Mini Kit (Qiagen). mRNA extracted from the salt-stressed oat seedlings after different durations was prepared for cDNA synthesis. The quality of the total RNA, mRNA, and final double-stranded cDNA samples was determined with an Agilent 2100 Bioanalyzer (Agilent Technologies). Five microgram samples of purified mRNA from the control and osmotically stressed samples were used as the templates for the synthesis of high-quality double-stranded cDNA. Each cDNA sample was then sheared via nebulization into small fragments and sequenced on the Illumina HiSeq 4000 platform. For each independent experimental replicate, approximately five plants were pooled for RNA preparation. A total of 12 RNA samples with three biological replicates each were prepared for transcriptomic analysis. The transcriptome datasets are available at the NCBI Sequence Read Archive (SRA) under accession number PRJNA354579.

### *De novo* assembly of cDNA sequences and functional annotation

Prior to assembly, the raw sequence reads were filtered to obtain high-quality clean reads. Adaptor sequences, low-quality nucleotides, and other contaminants were trimmed using a custom PERL script. After screening and trimming, the retained high-quality reads were assembled using Trinity with the default parameters. Functional annotation of the assemblies was performed by sequence similarity searches using the BLAST program against the NCBI nucleotide sequence database (nt), the non-redundant protein sequence database (nr), and the UniProt (Swiss-Prot) database with an E-value threshold of 10^−5^ ^14^. To identify the best BLASTx hits from the alignments, putative gene names, coding sequences, and predicted proteins were generated for the corresponding assembled sequences. Gene Ontology (GO) classification^15^ was performed based on the best BLASTx hits from the nr database using BLAST2GO software^16^ according to the molecular function, biological process, and cellular component ontologies with an E-value threshold of 10^−5^. The unigene sequences were also aligned to the COG database for functional prediction and classification, and they were annotated with Kyoto Encyclopedia of Genes and Genomes (KEGG) pathway information using the KAAS-KEGG Automatic Annotation Server online^17^.

### Differential expression analyses

For differentially expressed gene (DEG) analysis of the response of the two oat cultivars to salt stress, the reads per kilobase of transcripts per million mapped reads (RPKM) values from three replicates were calculated for each transcript in both cultivars. The significance of the difference in gene expression levels under salt stress was determined using DESeq, an R package program^18,19^. The false discovery rate (FDR) was applied to identify the P-value threshold in multiple tests^20^. Unigenes in which the FDR was <0.05 and the log2 ratio was >1 (two-fold change) between the two accessions were considered to be differentially expressed.

### Confirmation of expression profiles by qRT-PCR

To verify that the gene transcripts were differentially expressed between the two oat cultivars in response to salt stress, qRT-PCR analysis was performed on 18 unigenes that were identified as being differentially expressed based on the RPKM values. Total RNA was extracted from salt-stressed oat plants using the RNeasy Plant Mini Kit (Qiagen), and residual genomic DNA was removed by digestion with RNase-free DNase I (Promega) at 37 °C for 15 min. The RNA was then purified by phenol extraction, and 1 μg samples of purified total RNA from the two oat cultivars after various durations of salt stress were reverse transcribed into cDNA using an oligo d(T)_15_ primer (Promega) and SuperScript III reverse transcriptase (Life Technologies). The cDNA products were diluted 10-fold with nuclease-free deionized water prior to use as a template in qRT-PCR. qRT-PCR was performed in triplicate using the Platinum SYBR Green qRT-PCR SuperMix-UDG Kit on an ABI 7500 Real-Time PCR System (Life Technologies) according to the manufacturer’s recommendations. The thermal cycling conditions were as follows: 95 °C for 10 min followed by 40 cycles of 95 °C for 15 s and 60 °C for 1 min. The housekeeping gene actin was used as an internal control for normalization to allow comparison of the relative gene expression levels between the two accessions. The primers were designed using the Primer-Express program (Applied Biosystems) and are listed in Supplemental Table 1. To determine the relative fold differences for each sample in each experiment, the Ct value for each gene was normalized to the Ct value of the actin gene, and the difference between cultivars was calculated using the 2^−ΔΔCt^ method^21^. The specificity of amplification was verified at the end of each PCR run using the 7500 System SDS Dissociation Curve Analysis Software.

### Ethics approval and consent to participate

This manuscript does not report on or involve the use of any animal or human data or tissues, and therefore ethics problems are not applicable.

## Results

### Physiological differences between salt-sensitive and salt-tolerant oat genotypes in their response to salt stress

**Figure 1:**
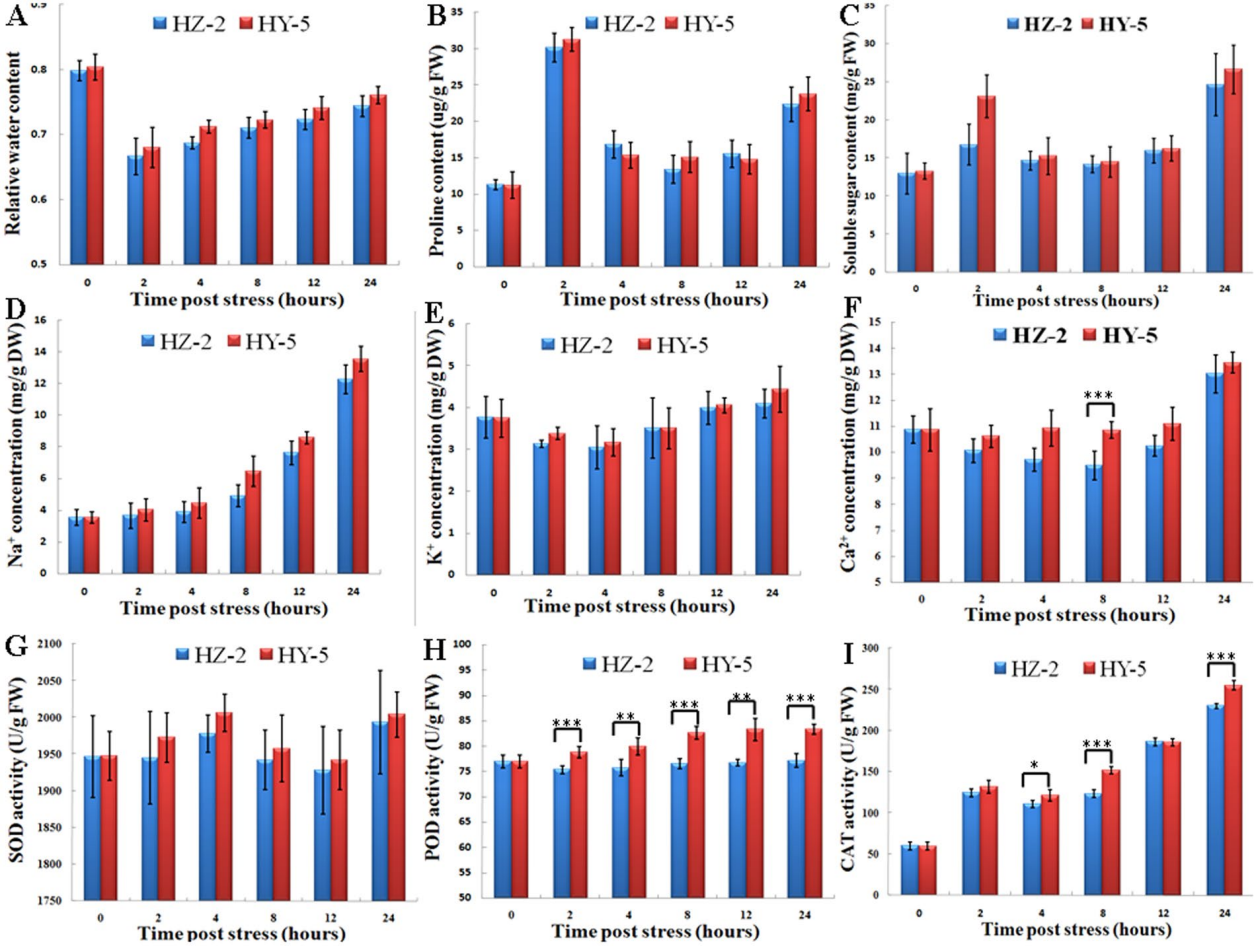
Physiological changes in ‘Huazao-2’ (HZ-2) and ‘Hanyou-5’ (HY-5) in response to various durations of osmotic stress. (**A**) Relative water content (RWC) levels; (**B**) Proline content; (**C**) Soluble sugar content; (**D**) Na^+^ concentration; (**E**) K^+^ concentration; (**F**) Ca^2+^ concentration; (**G**) Superoxide dismutase (SOD) activity; (**H**) Peroxidase (POD) activity; (**I**) Catalase (CAT) activity in seedlings treated with 100 mM NaCl. Data represent the means ± SE of three independent experiments; *P < 0.05; **P < 0.01; ***P < 0.001.

The response to osmotic stress by seedlings of the two genotypes was evaluated based on RWC, ion (Na^+^, K^+^, and Ca^2+^) contents, and the activities of reactive oxygen species (ROS) scavenging and detoxifying enzymes. The results of the physiological experiments are shown in Fig. 1. Two-way ANOVA analysis showed that compared with their values under normal growth conditions, the detected physiological indicators exhibited obvious changes in both cultivars (P < 0.01). However, the physiological changes at different time points differed between the two cultivars. Statistical analysis reveals significant differences in some physiological indicators, such as RWC (P-value 0.0022) and POD activity (P-value 0.0072), between the two cultivars after salt treatment, whereas no significant differences in physiological indicators were detected between the two cultivars under normal growth conditions. For example, the RWC of both cultivars declined rapidly within 2 h and then gradually recovered, but the RWC in ‘Hanyou-5’ was significantly higher than that in ‘Huazao-2’. However, up to 24 h, the RWC of both cultivars under salt stress was still lower than in the untreated control seedlings. With respect to osmotic adjustment, both cultivars showed approximately 4-fold increases in Na^+^ concentration within 24 h. Regarding antioxidant-related enzyme activities, in response to osmotic stress, SOD activity in both cultivars increased rapidly, peaked slightly after 4 h, then decreased in the next 12 h to reach the lowest level observed in this study before increasing again. CAT enzyme activity showed a similar trend but started to increase again after only 4 h. In particular, highly significant (P < 0.001) differences in both POD and CAT activities were observed between the two genotypes.

### *De novo* assembly and annotation of assembled transcripts

To provide a general overview of the gene expression profiles of the two oat cultivars, we used the Illumina HiSeq 4000 platform to sequence the salt-tolerant and salt-sensitive cultivar transcriptomes from osmotically stressed and unstressed seedlings of each cultivar at different time points. After filtering out adaptor sequences, low-quality reads, and ambiguous reads, a total of 378,437,410 and 383,183,792 reads remained from ‘Huazao-2’ and ‘Hanyou-5’, respectively, and were used for de novo sequence assembly. The de novo assembly of the high-quality cleaned reads from ‘Huazao-2’ generated 465,630 transcripts with an average length of 922 bp. For ‘Hanyou-5’, the assembly generated 460,234 transcripts with an average length of 931 bp. To obtain a more complete and representative picture of the naked oat transcriptome, the reads from all libraries were merged to form a combined dataset containing a total of 166,326 unigenes with an average length of 1,310 bp. An overview of the sequencing and assembly data is presented in Supplemental Table 2. All unigene sequences obtained in this study can be accessed as a fasta file (Supplemental File 1).

All assembled high-quality oat unigenes were annotated through homology searches against nucleotide and protein databases with a cut-off E-value of 10^−5^. Under these criteria, of the 166,326 unigenes, approximately 68.36% (113,704), 66.89% (111,266), and 50.09% (83,327) had significant hits in the GenBank nucleotide sequence database (nt), the non-redundant protein sequence database (nr), and the Swiss-Prot database, respectively. In terms of homology with sequences from different species, the majority of hits (28.33%) were found against stiff brome (*Brachypodium distachyon*), followed by winter barley (*Hordeum sativum*; 20.88%), barbed goatgrass (*Aegilops squarrosa*; 13.98%), and red wild einkorn (*Triticum urartu*; 8.34%). These annotation results show that most of our sequenced transcripts share close homology with mRNAs from other graminaceous species. Therefore, the assembled unigenes and the resulting annotation provide a reliable and comprehensive reference for research on the mechanisms of the transcriptomic response to salt stress in oat.

### Analysis of potential DEGs in the two oat cultivars

**Figure 2:**
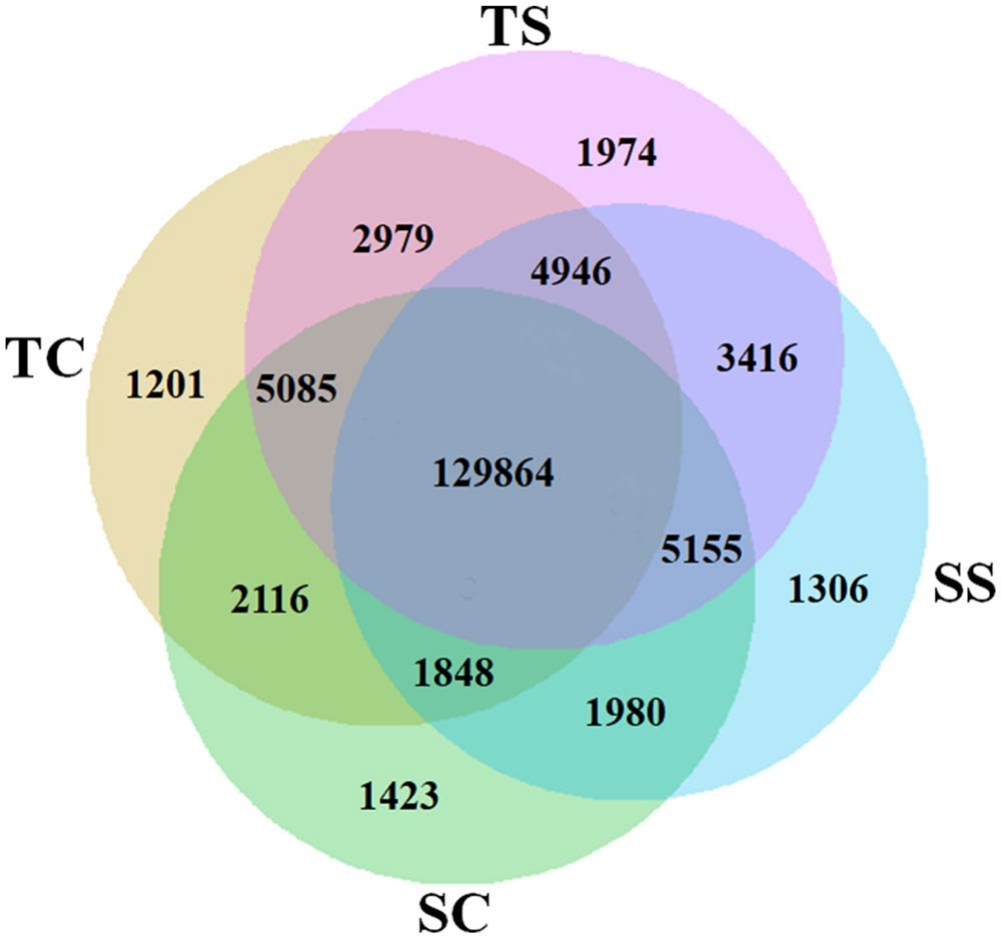
Statistics of differentially expressed genes under salt stress and normal growth conditions in the two genotypes of naked oat. The Venn diagram shows the number of differentially expressed genes between the two cultivars under salt stress treatment and normal growth conditions. Numbers of assembled transcripts in the salt-tolerant cultivar ‘Hanyou-5’ under salt stress (TS) and under control conditions (TC). Numbers of assembled transcripts in the salt-sensitive cultivar ‘Huazao-2’ under salt stress (SS) and under control conditions (SC).

To obtain an overall view of the genotype-specific salt-responsive gene expression in the two cultivars, RNA samples were prepared from osmotically stressed and control seedlings of ‘Hanyou-5’ and ‘Huazao-2’ at different time points. Analysis of the assembled unigenes shows that the expression of 21.92% (36,462/166,326) of the assembled unigenes was differentially regulated in the two cultivars at different durations of osmotic stress. The details of the statistics of the DEGs between the twelve independent treatments are shown in Fig. 2.

Further analysis of the gene expression profiles of ‘Hanyou-5’ and ‘Huazao-2’ under salt treatment and normal growth conditions was carried out by calculating the RPKM value of each unigene. Comparison of the RPKM values between the two cultivars for the control and salt treatments showed that the expression levels of 26,145 and 14,260 unigenes differed significantly (>2-fold, FDR < 0.05) between cultivars in response to osmotic stress and under normal conditions, respectively. The alterations included 16,529/9,616 genes that were up/down-regulated and 1,989/12,271 that were up/down-regulated by at least 2-fold under osmotic stress conditions and in the control, respectively. These genes included salt sensor or signal transduction genes, salt-responsive transcription factor genes, ion transport genes, and genes encoding detoxification enzymes (Supplemental File 2).

To precisely identify the specific biological processes that differed between the two cultivars, the assembled transcripts were further annotated with GO terms, COG categories, and KEGG pathway information. The number of unigenes in most of the GO categories increased in both cultivars in response to salt stress, but comparisons between the two cultivars showed much more complex and interesting results (Supplemental File 3). In some categories, the number of unigenes increased in both cultivars, but the salt-tolerant cultivar ‘Hanyou-5’ showed a greater increase than did the salt-sensitive cultivar ‘Huazao-2’. For example, in the Biological Process GO domain, the number of unigenes expressed in ‘Hanyou-5’ increased from 664 to 1,443 within 2 h, while the number of unigenes in the metabolic process category with lower expression levels in ‘Hanyou-5’ decreased from 6,382 to 247. With increasing treatment time, the number of unigenes with higher levels of expression increased continuously to 3,501, and the number with lower expression levels also increased to 1,136 by 24 h, indicating that under both normal growth conditions and stress conditions, metabolic-related gene expression differs greatly between ‘Hanyou-5’ and ‘Huazao-2’. In the Molecular Function GO domain, the number of unigenes with higher levels of expression in ‘Hanyou-5’ increased from 381 to 1,349 within 2 h, while the number of unigenes in the catalytic activity category with lower levels of expression in ‘Hanyou-5’ decreased from 4,948 to 200. With increasing time, the number of unigenes with higher expression levels increased continuously to 2,914, and those with lower expression levels also increased to 870 by 24 h of treatment, indicating that ‘Hanyou-5’ has higher levels of catalytic activity than ‘Huazao-2’ in response to osmotic stress. The differences that we detected suggest that the two oat cultivars might have developed different genetic pathways for adapting to salt stress.

### Pathway enrichment analysis of DEGs in the salt-tolerant oat cultivar ‘Hanyou-5’

Pathway enrichment analysis is an effective method for elucidating the biological functions of differentially expressed unigenes. Pathway-based analyses can aid in the identification of significantly enriched metabolic pathways and signal transduction pathways in DEGs by comparison with the whole-genome background expression levels. Our pathway enrichment analysis showed that the largest numbers of unigenes were in “metabolic pathways” followed by “RNA transport” in both cultivars and under both normal growth and osmotic stress conditions (Supplemental File 4). The metabolic pathways are large complexes associated with metabolic processes, such as the biosynthesis of secondary metabolites and carbohydrate, lipid, and amino acid metabolism. Comparison of the two cultivars by pathway enrichment analysis revealed that some pathways in the salt-tolerant cultivar ‘Hanyou-5’ contain many more members under salt stress conditions. For example, in the “plant-pathogen interaction pathway” category, the number of unigenes in ‘Hanyou-5’ increased by 10.57% under salt stress, while the number of members in ‘Huazao-2’ increased by 2.70%, a much smaller change than in ‘Hanyou-5’. Similar results were also found for ABC transporters, plant hormone signal transduction, the GnRH signalling pathway, metabolic pathways, the biosynthesis of secondary metabolites, and the TGF-beta signalling pathway. In addition, the number of unigenes in some pathways increased under salt stress in the salt-tolerant cultivar ‘Hanyou-5’ but decreased in the salt-sensitive cultivar ‘Huazao-2’. For example, in ‘Hanyou-5’, the number of unigenes in the oxidative phosphorylation category increased by 6.09% under salt stress, while in ‘Huazao-2’, the number decreased by 7.44%. Similar results were found in the MAPK signalling pathway, peroxisome, Toll-like receptor signalling pathway, and Wnt signalling pathway. The differences between the two cultivars with respect to pathway genes indicate that these pathways could play important roles in the response of oat seedlings to osmotic stress.

### qRT-PCR validation of differentially expressed transcripts from RNA-seq

**Figure 3:**
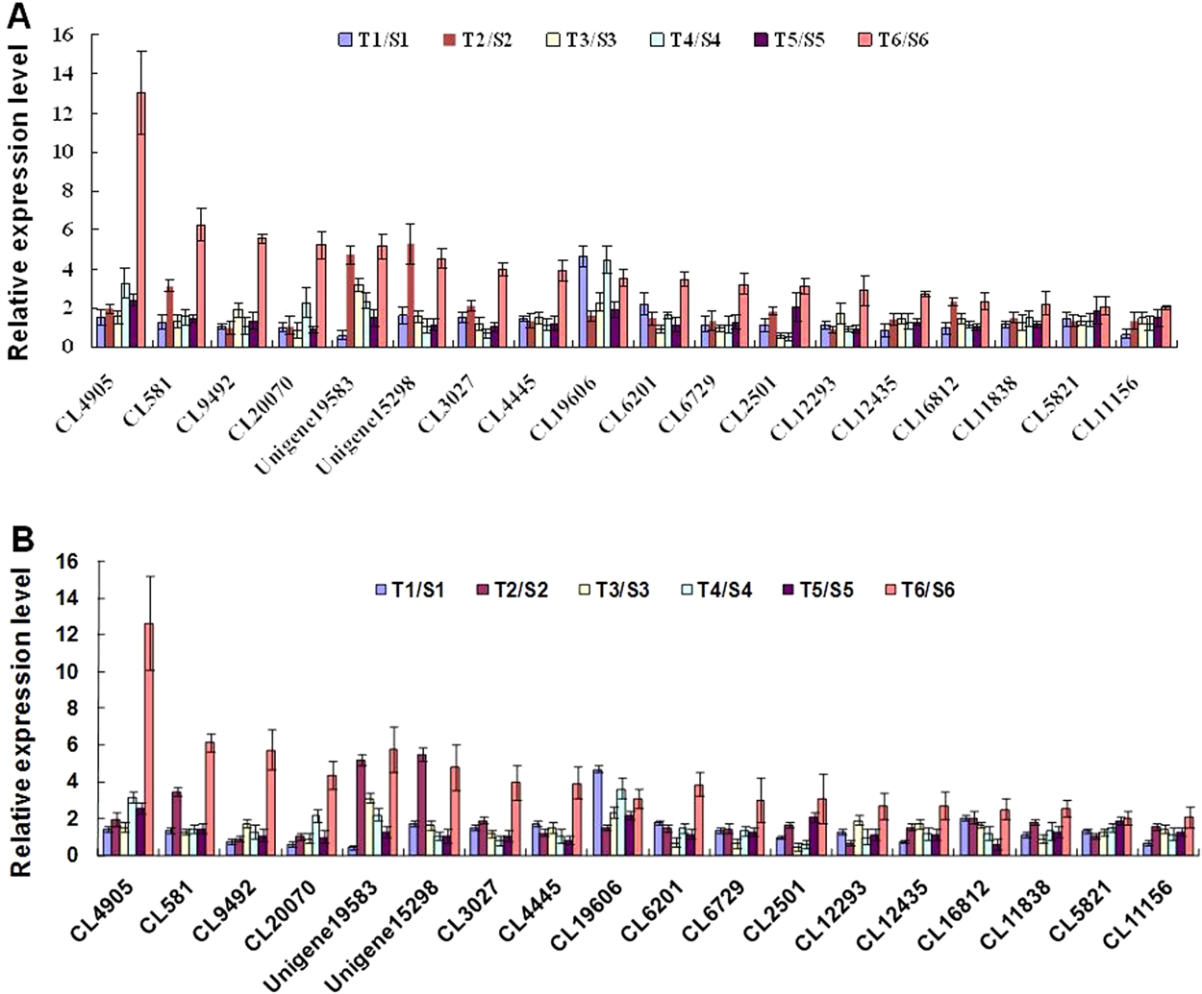
Validation of the expression patterns of selected oat genes by qRT-PCR. (**A**) Changes in the relative mRNA levels of 18 selected genes as determined by RNA-Seq. The Y axis indicates the relative fold change in transcript abundance under conditions of salt stress in relation to the control. S: ‘Huazao-2’ under normal growth conditions (1) and after 2 (2), 4 (3), 8 (4), 12 (5), and 24 (6) h of salt stress treatment. T: ‘Hanyou-5’ under normal growth conditions (1) and after 2 (2), 4 (3), 8 (4), 12 (5), and 24 (6) h of salt stress treatment. (**B**) Changes in the relative mRNA levels of 18 selected genes as determined by qRT-PCR. The qRT-PCR experiment was carried out with three biological replicates, and the actin gene was used as an internal control. The results are presented as target/reference ratios normalized by the internal control.

To further validate the NGS results, we conducted qRT-PCR analysis of selected metabolic pathway-related DEGs and calculated the relative expression levels of these genes using the 2^−∆∆Ct^ method. Although the fold changes determined by the two methods were not identical, the expression trends for all 18 genes were largely consistent between the qRT-PCR and RNA-seq analyses, demonstrating the overall reliability of the RNA-seq results (Fig. 3).

## Discussion

### Genotype-specific physiological responses to osmotic stress

Previous studies have shown that osmotic adjustment and ROS scavenging and detoxifying systems play important roles in osmotic stress responses^22^. In this study, we determined the changing profiles of osmolyte contents and the activities of antioxidant-related enzymes such as superoxide dismutase, catalase, and ascorbate peroxidase. The results of the physiological experiments indicate that although the two oat genotypes show similar trends in the early stages of their response to osmotic stress, the extent of physiological change is higher in ‘Hanyou-5’ than in ‘Huazao-2’. These results suggest that there is a common osmotic response mechanism between the two oat genotypes and that the differences in their osmotic stress response are largely a matter of degree. Compared with salt-sensitive ‘Huazao-2’, ‘Hanyou-5’ has a higher RWC, accumulates more Na^+^, K^+^, Ca^2+^ and has higher activities of antioxidant enzymes. Moreover, among these physiological indices, significant differences in the relative activities of POD and CAT were detected between the two genotypes, indicating that ROS scavenging and detoxification systems may play an important role in the osmotic stress response in oat.

### Construction of an informative transcriptome dataset for osmotically stressed oat seedlings

Salinity is one of the most important factors limiting the productivity of agricultural crops. Plant growth responds to salinity in two phases: a rapid osmotic phase that inhibits the growth of young leaves and a slower ionic phase that accelerates senescence in mature leaves^8^. Osmotic stress tolerance has a significant influence on the ability of plants to adapt to salinity and is an important component of the plant response to salt stress^23^. The decreased rate of leaf growth after an increase in soil salinity is primarily due to the osmotic effect of the salt on the roots. The breeding of salt-tolerant crop varieties that can be grown on marginal soils already affected by salinity is an effective approach. Long-term domestication and cultivation have generated many highly salt-tolerant crop germplasm resources. Previous studies have shown that different oat varieties have different levels of osmotic stress tolerance. However, the molecular mechanisms that control osmotic stress tolerance at the whole-plant level remain largely unknown. Molecular genetics and functional genomics provide new opportunities to synthesize molecular and physiological knowledge for the improvement of salt tolerance in plants. Because of their complexity, the genetic mechanisms that control salt tolerance in oat are not yet fully understood. Thus, elucidating the molecular genetic basis of the osmotic stress response in different oat cultivars will facilitate a better understanding of osmotic stress tolerance mechanisms and be an effective complement to enhancing tolerance to abiotic stress in oat.

To identify salt stress-tolerant oat cultivars, we previously screened 278 lines collected from different ecological zones in China by measuring their phenotypic responses under salt stress conditions, which proved to be an excellent strategy for salt stress tolerance research in oat^5^. Based on the screening results, the salt-tolerant cultivar ‘Hanyou-5’ and the salt-sensitive cultivar ‘Huazao-2’ were chosen for further analysis by high-throughput transcriptome sequencing under control and osmotic stress conditions using the Illumina HiSeq 4000 instrument. Further analyses of the assembled transcripts indicate that the differences in gene expression between the two cultivars may account for some of the observed differences in osmotic stress tolerance. Selecting expressed genes based on GO terms related to stress response highlights a potential starting point for understanding the underlying mechanisms. Several of these genes have been previously studied as they relate to stress tolerance. For example, functional analysis showed that many DEGs were enriched in GO terms such as “transporter activity”, “biological regulation category”, and “metabolic process category”. This information will be useful for elucidating osmotic stress tolerance mechanisms and for identifying new osmotic stress-related genes specific to naked oat. KEGG pathway analysis revealed that the DEGs were significantly enriched in metabolic pathways and signal transduction pathways. These up-regulated pathways included ABC transporters, oxidative phosphorylation, the Toll-like receptor signalling pathway, the MAPK signalling pathway, the calcium signalling pathway, peroxisome and plant hormone signal transduction, as well as other secondary metabolite pathways, suggesting that a rapid response to osmotic stress, maintaining osmotic balance, and eliminating the damage caused by oxygen stress may play vital roles in the expression of osmotic stress tolerance in oat.

### Molecular signals and their roles in transcription

Stress perception and signalling is the first step in the plant response to osmotic stress at the molecular level. Generally, the stress signal transduction pathway starts with signal perception, followed by the generation of secondary messengers, such as the modulation of intracellular Ca^2+^ levels, synthesis of hormones, or initiation of a protein phosphorylation cascade that ultimately targets proteins directly involved in regulating the transcription factors that control specific sets of stress-regulated genes. Recent studies have shown that stress sensing and signalling components can play important roles in regulating the expression of salt tolerance genes and transcription factors, and the overexpression of such genes in many plants resulted in improved stress tolerance^24^.

In our study, analysis of the DEGs between ‘Huazao-2’ and ‘Hanyou-5’ showed that the annotations “signalling process” and “signalling” in the GO biological process domain were highly enriched in the DEGs in response to osmotic stress. Pathway functional enrichment analysis also revealed that signalling pathways, such as calcium signalling and MAPK signalling, were significantly affected by the osmotic stress treatment. For example, multiple genes encoding proteins involved in stress signalling cascades, such as different types of protein kinases and phosphatases, Ca^2+^ messenger system proteins, and ROS scavenging systems and ABA signalling proteins, were detected in the salt-stressed mRNA library. Our data showed that the expression of MAPK genes (CL417, CL796, CL5690, CL7949, CL11923, and CL20367), serine/threonine-protein kinase genes (CL5127, CL9140, CL11407, CL18413, CL19001, Unigene24657, Unigene28302, and Unigene42012), and calcium-dependent protein kinase CDPK genes (CL73, CL8443, and Unigene28573) was greatly increased under osmotic stress in ‘Hanyou-5’, reflecting the multiplicity of osmotic stress signals in this salt-tolerant oat cultivar. Moreover, the increased activities of multiple signalling pathways are associated with the differential expression of various families of transcription factors, including zinc finger proteins, MYB, WRKY, NAC domain proteins, bzip transcription factors, and AP2 domain-containing transcription factors^25^. A previous study showed that the expression of some members of these transcription factor gene families responds to different types of stresses^26^. The differentially expressed signal transduction-related genes, including transcription factors, indicate that the difference in osmotic tolerance may be at least partly due to differences in the initial perception of salinity and the subsequent long-distance cascade signalling, or possibly in the efficiency of the signal transduction cascade^27^. Many unknowns remain in this area of osmotic stress research, and further investigations into the functions of these genes and metabolic pathways will help us to obtain a better understanding of stress tolerance.

### ROS scavenging plays a key role in the osmotic stress response in oat

Compared with the salt-sensitive cultivar ‘Huazao-2’, the salt-tolerant cultivar ‘Hanyou-5’ expressed many more genes related to antioxidant activity in the Molecular Function ontology, which implies that these antioxidant enzymes might play important roles in the tolerance to osmotic stress in oat. Abiotic stresses such as salinity can increase the production of reactive oxygen species (ROS), which were initially thought to be toxic by-products of aerobic metabolism but are now known to be central players in the complex signalling network of cells and have been shown to be important for regulating plant responses to many abiotic stresses^28^.

In osmotically stressed oat seedlings, many mRNA transcripts related to ROS scavenging enzymes were found to be differentially regulated during the acclimation to osmotic stress. Some of these, including multiple peroxidases (CL2154, CL2921, CL2921, CL2921, CL6892, CL7660, CL10001, CL15937, CL17696, CL19770, Unigene5637, Unigene8114, Unigene9183, Unigene12634, Unigene17988, and Unigene33859), reductases (CL2641, CL9939, CL9939, CL14731, CL20380, Unigene19874, Unigene25470, Unigene29251, and Unigene34495), superoxide dismutase (CL16854), and glutathione S-transferases (CL6036, CL18197, and Unigene25362), were found to be expressed at higher levels in ‘Hanyou-5’ than in ‘Huazao-2’. This finding suggested that ‘Hanyou-5’ probably has a more robust ROS scavenging system than ‘Huazao-2’ and that this system can be enhanced to increase the relative salt tolerance. Previous studies have indicated that these genes help to alleviate oxidative damage. For example, as the first line of defence against oxidative damage, SODs are usually induced by salinity to rapidly dismutate O_2_^−^ into H_2_O_2_ and oxygen, which are subsequently removed by various hydrogen peroxide decomposing proteins such as catalases and peroxidases^29^. The overexpression of SOD genes in other plants confers tolerance against salt and oxidative stresses^30^. POD is another enzyme that plays an important role in ROS scavenging. Previous studies have shown that the expression and activity of some POD enzymes are regulated in response to stresses and that these POD responses are directly involved in the protection of plant cells from adverse environmental conditions^31^. In *Jatropha curcas* L., a novel APX gene was cloned and found to be up-regulated by NaCl stress. Transgenic studies showed that plants overexpressing APX had increased tolerance to salt during seedling establishment and growth. Moreover, when subjected to 400 mM NaCl stress, the transgenic lines showed higher chlorophyll content and APX activity than the WT, which resulted in lower H_2_O_2_ content, indicating that the increased APX activity in the chloroplasts of the transgenic plants increased salt tolerance by enhancing ROS scavenging capacity under short-term NaCl stress conditions^32^. In this study, higher expression levels of genes in the antioxidative defence and ROS scavenging systems may help alleviate osmotic stress in oat by improving the overall antioxidative defence and radical scavenging activity and may play an important role in the acclimation to osmotic stress in oat.

## Conclusion

The use of NGS technologies for transcriptome sequencing in species that lack a complete reference genome is an effective approach to gene discovery and the identification of mRNAs involved in specific biological processes. In this study, we present a high-quality transcriptome of *A. sativa* subsp. *nudisativa* seedlings grown under conditions of salt stress. This genomic resource was produced using the Illumina HiSeq 4000 DNA sequencing instrument and de novo cDNA contig assembly. The large differences observed by digital expression analysis and functional annotation of salt-sensitive and salt-tolerant oat lines revealed the diversity of the approaches used by oats to address the challenge of salt stress. These line-specific data, as well as the remaining transcriptome sequences, will be useful in understanding the molecular mechanisms of salt tolerance in oats and will provide candidate genes or markers that can be used to guide future attempts to breed salt-tolerant oat cultivars.

## Electronic supplementary material

Supplementary Information

File S1

File S2

File S3

File S4

## Data Availability

The transcriptome datasets are available from the NCBI Sequence Read Archive (SRA) under accession number SRR5097463-SRR5097474.
