# Supplementary material for: Comparative Transcriptional Profiling and Physiological Responses of Two Contrasting Oat Genotypes under Salt Stress
Source: Sci Rep. 2018 Nov 2;8:16248. doi: 10.1038/s41598-018-34505-5 (PMC6214910; doi:10.1038/s41598-018-34505-5)
Supplement: Supplementary file 1 — Supplementary Information [file 41598_2018_34505_MOESM1_ESM.docx]

**Comparative Transcriptional Profiling and Physiological Responses of Two Contrasting Oat Genotypes under Salt Stress**

Bin Wu^1^, Yarvaan Munkhtuya^1^, Jianjiang Li^2^, Yani Hu^1^, Qian Zhang^1^, Zongwen Zhang^1,^*

1. Institute of Crop Science, Chinese Academy of Agricultural Sciences (CAAS), No. 12. Zhongguancun South Street, Beijing 100081, China

2. Institute of Grain Crops, Xinjiang Academy of Agricultural Sciences, No. 403. Nanchang Road, Urumqi 830091, China

* Correspondence: zongwenzh@sina.com

# Supplementary Materials

File S1: **Fasta file containing the 166,326 assembled oat cDNA unigene sequences.** Sequences were generated de novo by assembling nearly 0.76 billion filtered, high-quality reads.

File S2：**Unigenes that showed significant differential expression between the two oat genotypes after different durations of salt stress.** Unigenes with absolute expression values of |log2Ratio|>1 and FDR<0.05 were identified as DEGs.

File S3: **Gene Ontology analysis of all assembled oat transcripts.** All assembled transcripts in the twelve libraries with the best BLAST hits were used to search the GO database and grouped separately into the three main GO domains.

File S4: **The numbers of unigenes involved in various KEGG pathways.** Assignment of predicted proteins to different KEGG pathways.

Table S1: **Oligonucleotide primers used in qRT-PCR experiments in this study.** Excel file containing a list of primers used for experimental RT-PCR gene expression validation.

| **Unigene ID** | **Primer F** | **Primer R** | **Size (bp)** | **Seq. Description** |
| --- | --- | --- | --- | --- |
| CL4905.Contig8_All | 5'-CGGTTTGCCGAGCATCTG-3' | 5'-TCAAGGAGATCCTGGCCAAT-3' | 61 | Serine/threonine-protein kinase CTR1 [*Triticum urartu*] |
| CL581.Contig2_All | 5'-GGCCAGCCGCATGATG-3' | 5'-GGCCCAGAGATAAGGTTTCCTT-3' | 59 | Putative receptor-like protein kinase [*Aegilops tauschii*] |
| CL9492.Contig10_All | 5'-CCAAGGTCATGACAGCCAAA-3' | 5'-CTTCACTGCGCAAGATGCAT-3' | 56 | Sodium/hydrogen exchanger[*Triticum aestivum*] |
| CL20070.Contig1_All | 5'-GGCTCAGCGTGCTGCATTA-3' | 5'-TTTGGCACATCGCAAGGA-3' | 60 | Calcium-dependent protein kinase 3 [*Aegilops tauschii*] |
| Unigene19583_All | 5'-ACCTGTACTTGGTGGCGATGT-3' | 5'-ACCTGCATCTTGACGCAGAGT-3' | 60 | Glutathione S-transferase 3 [*Brachypodium distachyon*] |
| Unigene15298_All | 5'-TGGCGATGCCAAATTTAGC-3' | 5'-CCAATCGCTCGCTCGAA-3' | 57 | bZIP transcription factor D [*Triticum aestivum*] |
| CL3027.Contig20_All | 5'-ACAGGTCCATGCACTGTGATG-3' | 5'-CAGGTCGGGCCAACACAT-3' | 69 | ABC transporter C family member 9 [*Triticum urartu*] |
| CL4445.Contig6_All | 5'-TCCTCGCAGCATCCTCACT-3' | 5'-GGCGGCGAGCTTTTCAA-3' | 58 | CBL-interacting protein kinase 26-like [*Brachypodium distachyon]* |
| CL19606.Contig2_All | 5'-CGGCACAAAGGCGATGTC-3' | 5'-AATCGCTGGGCAGAGCAA-3' | 54 | MADS-box transcription factor 4-like [*Brachypodium distachyon*] |
| CL6201.Contig1_All | 5'-AAGCCGACCCAGTGGAAGTT-3' | 5'-CCTGGCATTCGTTCCATGA-3' | 57 | Dehydration Responsive Element Binding protein [*Leymus chinensis*] |
| CL6729.Contig4_All | 5'-GGCCTTCGGCGTCAGAT-3' | 5'-GCACCACCACGGGAACTTA-3' | 55 | Transcription factor bHLH47 [*Triticum urartu*] |
| CL2501.Contig1_All | 5'-CGCCGCTGGGCTCAT-3' | 5'-ACGCATCACACCCGTTGAT-3' | 59 | Peroxidase 8 [*Triticum monococcum*] |
| CL12293.Contig1_All | 5'-AAGGCACGCCCAGAGTGTTA-3' | 5'-CTTGCCGCTGCTCTGTTTC-3' | 63 | Peroxidase 16-like [*Brachypodium distachyon*] |
| CL12435.Contig1_All | 5'-CGCCTTCACCACGGGATA-3' | 5'-ACTCCCTAGAACCCCCATCTG-3' | 60 | WRKY17 transcription factor [*Triticum aestivum*] |
| CL16812.Contig5_All | 5'-GCACTGTTTGCTGACCTTTCG-3' | 5'-ACAACCGTGATGAATGCAATCT-3' | 65 | Potassium transporter 18 [*Aegilops tauschii*] |
| CL11838.Contig1_All | 5'-CCTGAGTCACGCCGGATCT-3' | 5'-GGGCAATTCGAACGATTCA-3' | 55 | MYB-related protein [*Aegilops tauschii*] |
| CL5821.Contig2_All | 5'-GGGCTTCGTCTCACATGGA-3' | 5'-ACGATGACGGAGACGGTACAG-3' | 56 | Transcription factor bHLH35 [*Aegilops tauschii]* |
| CL11156.Contig4_All | 5'-CCCCGTTGCTGCTGTATTTC-3' | 5'-CAAGAGAGTTCAGGTTGCACTCA-3' | 59 | Dehydrogenase/reductase SDR family member 12 [*Triticum urartu*] |

Table S2: **Summary of transcriptome sequencing and assembly results.**

| **cDNA Library** | **Total bases (bp)** | **High-quality reads** | **Average length (bp)** | **Total Length(nt)** | **Number of contigs** | **Number of singlets** | **Number of unigenes** |
| --- | --- | --- | --- | --- | --- | --- | --- |
| Unstressed HY-5 | 9,708,093,600 | 64,720,624 | 926 | 70,701,595 | 33,914 | 42,424 | 76,338 |
| 2h after osmotic stressed HY-5 | 9,708,093,600 | 64,720,624 | 887 | 63,475,915 | 31,265 | 40,305 | 71,570 |
| 4h after osmotic stressed HY-5 | 9,404,478,600 | 62,696,524 | 921 | 71,063,656 | 34,242 | 42,939 | 77,181 |
| 8h after osmotic stressed HY-5 | 9,506,063,100 | 63,373,754 | 933 | 76,317,725 | 37,314 | 44,485 | 81,799 |
| 12h after osmotic stressed HY-5 | 8,700,821,700 | 58,005,478 | 943 | 79,272,799 | 38,049 | 46,036 | 84,085 |
| 24h after osmotic stressed HY-5 | 9,738,060,900 | 64,920,406 | 923 | 68,905,671 | 32,902 | 41,755 | 74,657 |
| Unstressed HZ-2 | 9,505,184,400 | 63,367,896 | 925 | 63,623,729 | 29,363 | 39,448 | 68,811 |
| 2h after osmotic stressed HZ-2 | 9,820,997,700 | 65,473,318 | 877 | 57,841,699 | 27,451 | 38,467 | 65,918 |
| 4h after osmotic stressed HZ-2 | 9,484,960,800 | 63,233,072 | 905 | 61,873,697 | 28,422 | 39,931 | 68,353 |
| 8h after osmotic stressed HZ-2 | 9,708,093,600 | 64,720,624 | 950 | 78,650,739 | 37,280 | 45,529 | 82,809 |
| 12h after osmotic stressed HZ-2 | 9,250,238,700 | 61,668,258 | 957 | 83,781,323 | 40,127 | 47,439 | 87,566 |
| 24h after osmotic stressed HZ-2 | 9,708,093,600 | 64,720,624 | 972 | 84,375,262 | 40,021 | 46,756 | 86,777 |
| Combined samples | 114,243,180,300 | 761,621,202 | 1,310 | 217,953,515 | 98,129 | 68,197 | 166,326 |
